# AMY109, an interleukin-8 antibody, as a potential target for endometriosis treatment: a commentary

**DOI:** 10.1016/j.xfre.2025.08.003

**Published:** 2025-08-11

**Authors:** Taylor R. Wilson, Katherine A. Burns

**Affiliations:** Division of Environmental Genetics and Molecular Toxicology, Department of Environmental and Public Health Sciences, University of Cincinnati College of Medicine, Cincinnati, Ohio

## Interleukin-8 function

Interleukin-8 (IL-8) is a proinflammatory cytokine released during infections, inflammation, and tissue damage. Acting as a potent chemoattractant, IL-8 attracts inflammatory cells such as neutrophils and macrophages to sites of injury. Furthermore, IL-8 is often produced and released by a plethora of cells including neutrophils, macrophages, lymphocytes, endothelial, and epithelial cells. In addition to being proinflammatory, IL-8 is also a proangiogenic factor that promotes migration and growth of endothelial cells through matrix metalloproteinase production and the inhibition of endothelial cell apoptosis, which facilitates wound and tissue repair. In terms of function, IL-8 binds to G protein–coupled receptors, CXCR1 and CXCR2. CXCR1 has a higher specificity for IL-8, whereas CXCR2 has a higher affinity for IL-8. Both CXCR1 and CXCR2 are present on the cell surface of neutrophils, macrophages, and endothelial cells suggesting that they are likely targets for IL-8 therapeutics.

## IL-8 in endometriosis

In endometriosis, IL-8 has been a target of interest in understanding lesion development and disease severity for the past 3 decades. Studies evaluated potential mechanisms and quantitated levels of IL-8 in women with endometriosis. In the peritoneal fluid from women with endometriosis, the IL-8 levels increase compared with those in healthy controls and are significantly higher in the peritoneal fluid from women with more severe disease stages (1). Interleukin-8 is suggested to play a role in the attachment of endometrial stromal cells to fibronectin and treatment with IL-8 neutralizing antibody partially reduced endometrial cell adhesion, thus proposing that IL-8 plays a role in the attachment in lesion development. In the foundational work (2) to support this AMY109 study, the investigators found up-regulated expression of IL-8 in endometriotic lesions. To complicate potential therapeutic use of AMY109 in reducing the IL-8 levels, sex steroids such as estradiol and progesterone stimulate IL-8 production from endometrial endothelial cells in women with endometriosis demonstrating that the IL-8 secretion levels may be cycle stage dependent. To further complicate mechanistic studies to understand the role of IL-8 in endometriosis development in in vivo models, rodents do not produce IL-8; thus, nonhuman primate models or human samples are necessary to study potential mechanisms of IL-8 in endometriosis. In the foundational research (2) for this AMY109 study (3), once a month injection of AMY109 to primates with surgically induced endometriosis reduced lesion volume and ameliorated fibrosis and adhesions (2). Collectively, studies on IL-8 in endometriosis indicate that it may be a pivotal factor in the inflammatory environment having the ability to recruit inflammatory cells, attracting endothelial cells for vascularization, and supporting attachment of endometriotic lesions.

## Mechanistic action of AMY109

AMY109 is a long-acting anti–IL-8 monoclonal antibody developed to provide an alternative nonhormonal therapeutic for endometriosis. In nonhuman primates, this treatment produced no abnormal side effects except for reversible injection site reactions (2). These long-acting recycling antibodies, such as AMY109, are a more convenient treatment option than conventional antibody therapy because they are designed for prolonged exposure by binding and unbinding to the target receptor or antigen in a pH-dependent manner. The antibody binds in a neutral environment such as the bloodstream and then dissociates from the antigen in acidic environment (e.g., endosomes), which allows for binding to the neonatal fragment crystallizable receptor responsible for recycling antibodies back into the external cellular environment. The ability to recycle AMY109 internally in a pH-dependent manner increases the half-life of the antibody, conveniently, reducing the dose and the frequency of administration for the patient.

## Study limitations

Although AMY109 shows promising therapeutic potential, the clinical trial study (3) lacks key comparisons necessary to validate the therapeutic efficacy of AMY109. An efficacy concern for part 1 of the study design (i.e., safety in healthy volunteers) surrounds the large percentage of male participants (79.3%) and the lack of female participants included in the lower-dose groups (0.6- and 2.0-mg/kg AMY109). Although studying adverse side effects in both sexes is essential for the therapeutic to be used for nonfemale-specific diseases, the current focus for AMY109 is for women with endometriosis; therefore, the pharmacokinetic study population should reflect a higher percentage of women in the target population. Of note, sex-dependent differences in activation, maturation, and immunometabolism of neutrophils exist (4). Because these cells are a direct target of IL-8, we posit that the healthy volunteer population should reflect the target population to adequately evaluate AMY109 efficacy to avoid similar concerns found in other therapeutics with sex-dependent metabolic variances. An additional discrepancy exists around the age range for the recruited participants; the female volunteer maximum age was 10 years older than the male counterparts (65 years old for females vs. 50 years old for males). However, females in the postmenopausal age range (i.e., no hormonal cyclicity) will not be the intended demographic for AMY109 because these women are not actively menstruating and are not age-matched to the endometriosis treatment groups present in part 2. Furthermore, the population demographic is skewed toward Asian volunteers with no other ethnicity present in part 2, whereas part 1 had a more diverse population. In future studies, the population demographics will need to be increased to determine whether demographic specific differences in treatment exist.

The most common adverse side effects were various infections associated mostly with the respiratory and gastroenteric systems; however, these findings are a consequence of diminished immune cell response from the anti–IL-8 treatment. Interestingly, the investigators found that AMY109 did not disrupt the physiology of the menstrual cycle; however, ovulation and fertility studies will need to be evaluated in the future for reproductive safety. Especially of note, the healthy female volunteers in part 1 of the study had to be on a contraceptive, postmenopausal (i.e., not menstruating), or surgically sterile, which indicates that they were not evaluated for disruption of their menstrual cycles. Although the women in part 2 of the study were only patients with endometriosis with moderate to severe dysmenorrhea, they did not find cyclicity problems but also did not note cycle length or in which cycle stage the dose of AMY109 was given. Of importance to studying reproductive safety, during ovulation, the oocyte is released after follicular rupture as a result of increased inflammatory cytokines including IL-8 (5). Knocking down or blocking IL-8 in the peritoneal cavity may result in negative consequences on ovulation and further affect fertility in women with endometriosis; therefore, ovulation should be a priority to be evaluated in AMY109 clinical trials.

An additional limitation to the study is that the low dose of AMY109 in part 2 of the study does not match the low dose reported for safety in part 1 of the study (0.8 mg/kg in part 2 vs. 0.6 mg/kg in part 1). The safety data from part 1 are also not directly comparative to those from part 2 because the healthy volunteers received a single dose of AMY109, whereas the endometriosis group in part 2 received 6 doses (i.e., once a month) of AMY109 over 6 months of treatments. These comparisons may confound these findings/safety data because women with endometriosis already have higher levels of IL-8 and may tolerate higher doses of AMY109 treatment. Importantly, after surgery, inflammation will decline in women with endometriosis, and a higher dose of AMY109 may be too potent; however, further studies will be needed to address these concerns.

## Conclusion

Collectively, AMY109 is a potential promising therapeutic worthy of further consideration as a treatment for endometriosis. Although a phase 1 clinical trial should require a larger sample size and the inclusion of more female participants, this study suggests that AMY109 is a nonhormonal therapeutic for the treatment of endometriosis. A promising aspect of the study was the inclusion of pain metrics in women with endometriosis; AMY109 treatment required patients to take less analgesic pain medications for nonmenstrual and menstrual pelvic pain. The findings suggest that AMY109 treatment reduces pain in patients with endometriosis; however, a follow-up study will require increased sample sizes to validate whether the pain response is significant. Additionally, the study provides evidence that AMY109 has a good safety profile in males and females after 1–6 doses of the therapy. Worth mentioning, the 40-day half-life of AMY109 is a benefit for convenience for patients because this would decrease dose and frequency of administration. Overall, although the study by Wang e. al. (3) requires additional validation, AMY109 may serve as a future therapeutic option for the management of pain and treatment of endometriosis.

## CRediT Authorship Contribution Statement

**Taylor R. Wilson:** Writing – review & editing, Writing – original draft, Conceptualization. **Katherine A. Burns:** Writing – review & editing, Conceptualization.

## Declaration of Interests

T.R.W. has nothing to disclose. K.A.B. has nothing to disclose.
